# Reduction in unplanned hospitalizations associated with a physician focused intervention to reduce potentially inappropriate medication use among older adults: a population-based cohort study

**DOI:** 10.1186/s12877-021-02172-3

**Published:** 2021-03-31

**Authors:** M. Alcusky, R. B. Thomas, N. Jafari, S. W. Keith, A. Kee, S. Del Canale, M. Lombardi, V. Maio

**Affiliations:** 1 Department of Population and Quantitative Health Sciences, University of Massachusetts Medical School, Albert Sherman Building, 6th Floor, 368 Plantation Street, Worcester, MA USA; 2 Jefferson College of Population Health, Thomas Jefferson University, 901 Walnut St., 10th Floor, Philadelphia, PA 19107 USA; 3 Center for Research in Medical Education and Health Care, Sidney Kimmel Medical College, Thomas Jefferson University, Philadelphia, PA USA; 4 Division of Biostatistics, Department of Pharmacology and Experimental Therapeutics, Sidney Kimmel Medical College, Thomas Jefferson University, Philadelphia, PA USA; 5 Azienda Unità Sanitaria Locale di Parma (Local Health Authority of Parma), Parma, Italy

**Keywords:** Prescribing, Hospitalizations, Older adults

## Abstract

**Background:**

A multimodal general practitioner-focused intervention in the Local Health Authority (LHA) of Parma, Italy, substantially reduced the prevalence of potentially inappropriate medication (PIM) use among older adults. Our objective was to estimate changes in hospitalization rates associated with the Parma LHA quality improvement initiative that reduced PIM use.

**Methods:**

This population-based longitudinal cohort study was conducted among older residents (> 65 years) using the Parma LHA administrative healthcare database. Crude and adjusted unplanned hospitalization rates were estimated in 3 periods (pre-intervention: 2005–2008, intervention: 2009–2010, post-intervention: 2011–2014). Multivariable negative binomial models estimated trends in quarterly hospitalization rates among individuals at risk during each period using a piecewise linear spline for time, adjusted for time-dependent and time-fixed covariates.

**Results:**

The pre-intervention, intervention, and post-intervention periods included 117,061, 107,347, and 121,871 older adults and had crude hospitalization rates of 146.2 (95% CI: 142.2–150.3), 146.8 (95% CI: 143.6–150.0), and 140.8 (95% CI: 136.9–144.7) per 1000 persons per year, respectively. The adjusted pre-intervention hospitalization rate was declining by 0.7% per quarter (IRR = 0.993; 95% CI: 0.991–0.995). The hospitalization rate declined more than twice as fast during the intervention period (1.8% per quarter, IRR = 0.982; 95% CI: 0.979–0.985) and was nearly constant post-intervention (IRR: 0.999; 95% CI: 0.997–1.001). Contrasting model predictions for the intervention period (Q1 2009 to Q4 2010), the intervention was associated with 1481 avoided hospitalizations.

**Conclusion:**

In a large population of older adults, a multimodal general practitioner-focused intervention to decrease PIM use was associated with a decline in the unplanned hospitalization rate. Such interventions to reduce high risk medication use among older adults warrant consideration by health systems seeking to improve health outcomes and reduce high-cost acute care utilization.

**Supplementary Information:**

The online version contains supplementary material available at 10.1186/s12877-021-02172-3.

## Introduction

The danger of potentially inappropriate medications (PIMs) for older adults has been well-known to healthcare practitioners for the better part of the past few decades [1, 2]. The risks posed by PIMs are magnified as the number of medications older adults are exposed to rises [3], increasing the opportunity for adverse events related to drug interactions or errors in use [4]. The use of PIMs in older adults leads to worse clinical outcomes [5–8], higher healthcare resource utilization, such as increased hospitalization [9–11], and higher costs to the healthcare system as a whole [12, 13]. Some of the most common side effects of PIM use in older adults include falls, orthostasis, constipation, and acute kidney injury [14].

Reduction of PIM use has become a priority of healthcare systems. Many interventions to reduce PIM use in older adults have been piloted and implemented, and studies have shown them to be effective [15–17]. Though PIM use in older adults is associated with increasing hospitalizations [9–11], there is limited evidence on the effect that interventions to decrease PIM use in the older adult population have on hospitalization rates. A recent Cochrane review reported mixed results across five studies examining the effect of multifaceted pharmaceutical care-based interventions to improve medication use in older adults on hospitalizations [16]. Interventions varied from the use of explicit criteria when assessing patients to the inclusion of a pharmacist on the care team [16]. However, prior studies have generally been small and have not evaluated interventions to reduce PIM use targeted at the level of an entire local healthcare system, as in the current study.

We previously conducted a physician-focused, multimodal, quality improvement initiative to reduce PIM exposure in the older adult population of the Local Health Authority (LHA) of Parma, Italy [15, 18]. The initiative involved development of an Italian-focused PIMs list by an expert panel, multimodal education and dissemination of the list targeting all 303 general practitioners (GPs) in Parma LHA, and feedback on performance (i.e., PIM prescribing rates) to GPs over time. The intervention focused on GPs because they serve as gatekeepers and the primary managers of care for patients in Italy, and GPs have historically prescribed PIMs more often than specialists [19]. The value of an education-focused intervention for GPs in Parma LHA was supported by a pre-intervention survey that identified knowledge gaps regarding PIM prescribing for older adults [20]. In prior studies of the intervention’s association with changes in PIM use, we found the proportion of older adults exposed to PIMs declined 31.4% from the pre-intervention period (7.8%) to the intervention period (5.3%) [15], and the lower prevalence of PIM exposure was sustained even after the intervention’s termination [18]. However, it remains to be seen whether our intervention was associated with a decrease in hospitalizations. Due to the known association between PIM exposure and increased hospitalization rates [9–11], we hypothesized that our quality improvement intervention that successfully reduced PIM exposure [15] led to lower unplanned hospitalization rates. The objective of this study was to quantify changes in unplanned hospitalization rates among older adults in Parma LHA associated with a quality improvement intervention that reduced PIM exposure.

## Methods

### Setting

Italy consists of a single-payer healthcare system, in which the country is divided into 21 regional governments, and healthcare is delivered through networks of geographically defined LHAs. Each citizen is assigned a primary care physician or pediatrician within a specific LHA [21]. Italian residents are not permitted to opt out of the public system, so substitutive private coverage does not exist, although a small fraction (~ 10%) of residents purchase complementary insurance to cover co-payments or supplemental insurance to cover expanded services [22].

### Data source and structure

We conducted a retrospective longitudinal cohort study for the years 2005–2014 using the administrative healthcare database of the LHA of Parma, which covers approximately 420,000 inhabitants. The Parma LHA healthcare administrative database is comprehensive and includes de-identified demographic, hospital, outpatient, and pharmacy information for all covered members and services covered by the public system.

### Study population

Residents of the Parma LHA were included in the analysis for a given quarter (Q1: January 1–March 31, Q2: April 1–June 30, Q3: July 1–September 30, or Q4: October 1–December 31) if they were alive at the start of the quarter, were at least 65 years old at the beginning of the quarter, and remained residents of the LHA of Parma for the duration of the calendar year (residency data were only available annually). Residents were followed until death, out-migration from the LHA, long-term hospitalization of greater than one quarter, or the end of the study period.

### Intervention

The physician-focused intervention to reduce inappropriate prescribing among older adults in Parma LHA was implemented in stages from the fourth quarter of 2007 (Q4 2007) through Q4 2009. This intervention which engaged all 303 LHA GPs, is described in detail elsewhere [15]. In essence, the multimodal intervention was implemented in 3 stages. The first stage (2007 Q1-Q4) included the development of an Italian-focused PIMs list by an expert panel. This stage ended with dissemination of the list, along with PIM prevalence data for each district of Parma LHA, to GPs via email and through educational sessions using a peer-to-peer interactive approach. In stage 2 (2008 Q1-Q4), the expert panel developed an alternative list of drugs to substitute for PIMs, which was presented to GPs along with PIM prevalence data. In phase 3 (2009 Q1-Q4), the expert panel developed case studies based on the most prevalent PIM scenarios, which were then presented to the GPs along with PIM prevalence data [15]..

We anticipated a slight lag between the initiation of our quality improvement intervention to reduce PIM exposure, changes in prescribing practices, and the hypothesized association with unplanned hospitalizations. Reviewing the raw data confirmed our expectations and we applied a one-year lag between the start of the intervention and the beginning and end of the exposure period to account for this delay. Therefore, older adults were considered exposed to the PIM-focused QI initiative during the period Q1 2009 to Q4 2010. The pre-intervention period spanned Q1 2005 to Q4 2008 and the post-intervention period spanned Q1 2011 to Q4 2014. We performed two sensitivity analyses testing a shorter (0.5 year) and longer (1.5 years) lag period between the beginning of the quality improvement initiative and the intervention’s effect on hospitalization rates.

### Outcome

The primary study outcome was all-cause unplanned hospitalizations (i.e., those that were unscheduled and required an urgent level of care). We counted the number of individuals with an unplanned hospital admission during each quarter of our 10-year study in which they were at risk (alive and living in Parma LHA). To explore one possible mechanism by which the intervention may have reduced unplanned hospitalization rates, we also examined a composite secondary outcome of cause-specific hospitalizations for diagnoses representing potential adverse drug events associated with non-steroidal anti-inflammatory drugs (NSAIDs) and digoxin (Additional File 1). The intervention has previously been associated with large decline in NSAID and digoxin use in Parma, LHA [15].

### Covariates

Demographic characteristics included age, sex, and geographic location (plain, hill, or mountain). In Parma LHA, geographic location may be considered a proxy for population density with residence in either the plains, hills, or mountains roughly corresponding to urban, suburban, and rural areas. Pharmacy outpatient data were used to calculate the number of Chronic Condition Drug Groups (CCDGs) in the year prior [23]. As a time-dependent summary score for medical morbidity, CCDGs can identify up to 31 chronic conditions; the higher the CCDG score, the greater the number of chronic conditions. The CCDG score has been used in numerous studies as a comorbidity index [9, 24–26]. For the analysis, the CCDG score was updated in a sliding window every quarter.

### Statistical methods

Descriptive statistics were used to summarize the characteristics of the study population during the pre-intervention, intervention, and post-intervention periods. Crude hospitalization rates were calculated for each period as the average of the number of hospitalizations divided by the number of older adults at risk during each year of the period.

Multivariable longitudinal negative binomial generalized estimating equations (GEE) modeling was used to estimate trends in quarterly hospitalization rates (counts of hospitalizations over the number of eligible older adults) during the pre-intervention, intervention, and post-intervention periods using a piecewise linear truncated power basis spline for time. The model adjusted for correlation within individuals and for time-dependent and time-fixed covariates. Sex was considered as the only time-fixed variable in the model. The time dependent variables included: age at the start of each quarter, yearly updated geographic location, number of CCDGs in the previous four quarters, season, and three spline time trend variables. Time ranged from − 15 to 24 over 40 quarters of the study period with time 0 corresponding to the last quarter of the pre-intervention period (Q4 2008). Knots, the break points in the spline function where the pieces of the time trend were allowed to change, were placed at time 0 and at time 8 (Q4 2010). In the two sensitivity analyses to test a shorter and longer lag period between the start of the intervention and its effect on the outcome, the knots were shifted backward in time by two quarters (to test a shorter 0.5 year lag period) or forward by two quarters (to test a 1.5 year lag period).

For presentation of results, the spline parameters were combined to represent piecewise linear slopes on the log rate scale so that the anti-log of those quantities represented the expected incidence rate ratio changes associated with quarterly increases in time for each period. To estimate the number of hospitalizations potentially avoided by the intervention, we contrasted model predictions for the intervention period in the presence of the intervention (i.e., consistent with data observed) and in the absence of the intervention (i.e., projected, as though the intervention never happened). To estimate the number of hospitalizations that might have occurred absent the intervention, the trend modeled in the pre-intervention period was projected through the intervention period and the expected value for hospitalization counts was estimated and summed for each quarter of the intervention. This value was then compared with the number of hospitalizations for the intervention period as predicted by the model in the presence of the intervention (i.e., observed), and the difference was reported as the number of hospitalizations avoided.

Taking the perspective of the Parma LHA, we applied the median length of hospital stay values from our patient population and average cost-per-stay estimates for hospitalizations in Italy [27] to estimate the average cost of the hospitalizations avoided by the intervention during the intervention period. The average cost per inpatient bed day of a hospitalization in Italy has been estimated to be $506 (2010 USD; not including drug costs) [27], and the median length of stay in our patient population was 8 days. Therefore, each hospitalization was estimated to cost $4048.

## Results

The study population included 151,747 older adults corresponding to 973,927 person-years, of which 382,710 person-years (39.3%) were contributed to the pre-intervention period, 193,901 person-years (19.9%) to the intervention period, and 397,316 person-years (41%) to the post-intervention period. The majority of person-time was contributed by women, older adults ages 65–74 years, residents of plains regions, and those with 4 or more chronic conditions (Table 1). The fraction of person-time contributed by adults with 4 or more chronic conditions was 74.2% in the pre-intervention compared with 78.8% during the intervention and 79.6% post-intervention.

**Table 1:** Characteristics of Older Adults in Parma LHA (*N* = 151,747) during the Pre-Intervention, Intervention, and Post-Intervention Periods

	Pre-Intervention Q1 2005 – Q4 2008	Intervention Q1 2009 – Q4 2010	Post-Intervention Q1 2011 – Q4 2014
Number of Residents	117,061	107,347	121,871
Person-Years Contributed	382,710	193,901	397,316
Age Groups (%)^a^			
65–74	49.2	48.5	47.8
75–84	37.1	36	35.8
85+	13.7	15.5	16.4
Sex (%)^a^			
Women	58.8	58.3	57.7
Geographic Location (%)^a^			
Hill	31.1	31.2	31.6
Mountain	10.6	10.3	10
Plain	58.3	58.5	58.4
Number of Chronic Conditions (%)^a^			
0	11.9	10.2	10.2
1–3	13.8	11	10.2
4+	74.2	78.8	79.6
Hospitalizations per 1000 person-years	146.2	146.8	140.8

^a^Percentage represents proportion of person-years contributed

**Fig. 1:**
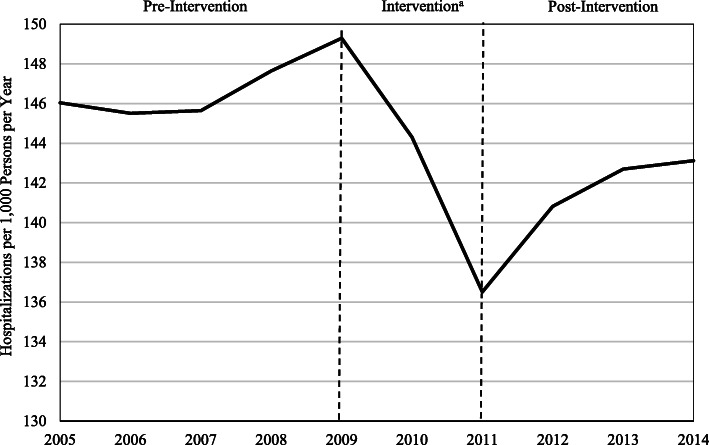
Crude Hospitalizations Rates during the Pre-Intervention, Intervention, and Post-Intervention Periods. ^a^The quality improvement initiative was implemented in stages beginning in the fourth quarter (Q4) of 2007 and ending in 2009 (Q4). The beginning and end of the intervention period reflects a one-year lag from the start and end of the intervention

Crude hospitalization rates for the pre-intervention, intervention, and post-intervention periods were 146.2 (95% CI: 142.2–150.3), 146.8 (95% CI: 143.6–150.0), and 140.8 (95% CI: 136.9–144.7) hospitalizations per 1000 persons per year, respectively. Crude hospitalization rates began increasing in the 2 years preceding the intervention period and then decreased during the intervention before increasing and leveling off during the post-intervention period (Fig. 1).

After adjusting for demographic characteristics, the pre-intervention hospitalization rate was estimated to have been declining by 0.7% each quarter (IRR = 0.993; 95% CI 0.991–0.995). Hospitalization rates during the intervention were estimated to have been declining by 1.8% each quarter (IRR = 0.982; 95% CI 0.979–0.985). Post-intervention hospitalization rates were estimated to have been declining by 0.1% each quarter (IRR = 0.999; 95% CI 0.997–1.001; Table 2). Associations were consistent in sensitivity analyses testing a shorter and longer lag period between the start of the intervention and its effect on hospitalization rates (Additional File 2). The association between the intervention period and the secondary composite outcome of potential NSAID and digoxin cause-specific hospitalizations was directionally consistent but weaker than the association with the primary outcome (Additional File 3).

**Table 2:** Association between a Quality Improvement Initiative to Reduce PIM Use and Quarterly Unplanned Hospitalization Rates

	Estimate^a^	IRR^a^ (95% CI)
Time (per quarter)^b^		
Pre-Intervention	−0.007	0.993 (0.991, 0.995)
Intervention	−0.018	0.982 (0.979, 0.985)
Post-Intervention	−0.001	0.999 (0.997, 1.001)

^a^Estimates and incidence rate ratios for quarterly changes in unplanned hospitalization rates during the specified time period from a multivariable longitudinal negative binomial GEE model

^b^Time represented in the model as the piecewise linear truncated power basis spline. The pre-intervention, intervention, and post-intervention estimates were calculated from the linear combination of coefficients for three time trend variables

*IRR* Incidence Rate Ratio, *CI* Confidence Interval, *PIM* potentially inappropriate medication

Associations between covariates and hospitalization rates are presented in Addtional File 4. The hospitalization rate was 30% lower among women (IRR: 0.700; 95% CI: 0.688–0.711), while each one-year increase in age was associated with a 6.4% higher (IRR: 1.064; 95% CI: 1.063–1.065) hospitalization rate.

Extrapolating the pre-intervention trend through the intervention period, an estimated 31,261 unplanned hospitalizations would have occurred absent the intervention. In the presence of the intervention, an estimated 29,780 unplanned hospitalizations occurred during the intervention period. Therefore, an estimated 1481 unplanned hospitalizations may have been avoided by the intervention. Applying the average cost of $4048 per unplanned hospitalization, our analyses suggest a potential savings of $5,995,088 from avoiding these unplanned hospitalizations over the 2 years of the intervention.

## Discussion

A regional physician-focused quality improvement initiative that successfully reduced PIM utilization was associated with the acceleration of a pre-existing trend of declining unplanned hospitalization rates among older adults in Parma LHA. The estimated avoidance of almost 1500 unplanned hospitalizations during the short two-year intervention period suggests the intervention may have led to clinically meaningful benefits to residents and substantial cost-savings of almost $6,000,000 to the healthcare system. In the context of an aging population and an increasing burden of multimorbidity in our population and internationally, these findings underscore the central role that appropriate medication management continues to play in the prevention of serious adverse events for high-risk older adults. As healthcare systems are increasingly held accountable for the quality and cost of care delivered, investment in system-level prescriber focused interventions to reduce PIM use among older adults should be considered by policymakers and organizational leaders as a promising avenue towards achieving these dual objectives.

Although the hospitalization rate leveled off after the intervention period, absence of an increasing trend post-intervention suggests that PIM prescribing practices did not revert to baseline levels, consistent with prior evaluation of the intervention’s long-term effect on PIM use [18]. The deceleration of the rate of decline in hospitalizations was likely the result of a few factors. Declining PIM use inherently presents fewer opportunities to intervene. Furthermore, individual PIMs and classes of PIMs vary substantially in the magnitude of increased hospitalization risk associated with exposure [9–11], and the ease with which deprescribing barriers can be overcome [28]. Once providers made initial changes in their clinical practice to reduce exposure to the most dangerous and most easily substituted medications, diminishing marginal returns can be expected as only PIMs deemed relatively less dangerous or difficult to avoid would remain as potential targets for regimen modification.

There is growing evidence to support an association between PIM exposure and hospitalization in older adults [9–11]. However, there has been relatively little research exploring the relationship between interventions to reduce PIM use in older adults and hospitalization rates. A 2015 review [16] identified 5 studies focused on interventions to improve polypharmacy in older individuals that also included hospitalizations as an outcome measure [29–33]. Previous studies have varied in structure and outcomes measured. The majority were focused on the effects of integrating a pharmacist into the healthcare team [30–33] and other interventions included incorporating medication reviews using explicit criteria [29], and implementation of a medication therapy management program [33]. Results of a recent meta-analysis of randomized controlled trials support the effectiveness of this type of pharmacist-led intervention. The odds of adverse drug reactions among older adults exposed to pharmacist-led interventions were 21% lower and the odds of serious adverse drug reactions were 36% lower than control arms [17].

To the best of our knowledge, the quality improvement initiative in Parma LHA was the first multiphase, multimodal educational outreach intervention targeting PIM reduction that has been delivered and evaluated at the level of an entire local health system. In contrast to most prior studies of PIM focused interventions which modified workflow and team structure within a limited clinical context, our study estimated changes in unplanned hospitalization rates for the full population of over 100,000 older adults residing in Parma LHA. Although the multifaceted nature of the intervention precludes estimation of effects for specific components, individual components have been previously examined. Studies of educational outreach visits (i.e., academic detailing) have reported small but consistent effects on prescribing, with mixed results for studies comparing educational visits delivered to individuals versus groups [34]. Continuing education meetings and workshops have also been found to precipitate small but potentially meaningful changes in clinical practice [35], with larger effects observed for interventions that involve interactive components, as was the case in Parma LHA. However, most earlier studies have stopped short of establishing a connection between changes in prescribing and health outcomes such as hospitalization.

Limitations of the present study stem primarily from the absence of a contemporary comparison group that was unexposed to the intervention. Although other secular changes affecting hospitalization rates during the intervention period cannot be excluded as potential confounders, no other system-wide interventions to reduce hospitalization rates were implemented in Parma LHA during the intervention period. Although we adjusted for the increasing age and morbidity burden observed in later time periods in our study, which led to the differences between crude and adjusted trends, the direction of residual confounding associated with the aging of the population is expected to bias results against an association with the intervention. Other secular trends included seasonal fluctuations in influenza severity and increases in socioeconomic stress associated with the economic recession that began in 2008. In the case of the former, influenza severity among older adults in the neighboring region of Lombardy, Italy, was greatest during the post-intervention period (2011–2014) [36]. The economic recession in Italy was most severe during the latter years of our study period (2012–2014) [37], and has been associated with increases in unhealthy behaviors [38] and increases in hospitalizations for myocardial infarction [39].

The selection of the lag period (one-year) represents a potential limitation, as the onset of the PIM education initiative’s effects on hospitalizations may have begun before 1 year into the intervnetion and may have continued beyond 1 year after the end of the initiative. After adjusting for increasing age and medical morbidity over time, hospitalization rates were declining during the pre-intervention period, and it is possible the first phase of the intervention (Q42007 to Q4 2008) contributed to this trend. Additionally, we studied changes in the rates of all-cause unplanned hospitalizations because of the low sensitivity with which iatrogenic hospitalizations can be identified using administrative data. Therefore, we were unable to distinguish between changes in PIM-induced hospitalizations and hospitalizations not caused by PIMs. Finally, our estimates of unplanned hospitalizations avoided and savings as a result of hospitalizations avoided are crude estimates and do not consider the cost of implementing the intervention. Our cost estimate was from the perspective of the LHA of Parma, where the cost of a hospitalization is on average half that of a hospitalization in the United States [27, 40]. Therefore, the cost savings associated with a similar number of hospitalizations avoided by a US based (or other high-cost) health system would be much larger.

Our study was conducted in a single LHA in Italy, but can reasonably be expected to be generalizable to other parts of the country. Other health systems considering a prescriber-focused intervention to reduce PIM use and associated hospitalizations should consider factors such as the baseline levels of PIM use and prescriber knowledge gaps to understand the size of the potential opportunity for improvement associated with such an intervention. Furthermore, an important component of our intervention’s success in Parma LHA was the high levels of engagement from the provider community. This was facilitated by the inclusion of GPs in the design and implementation of the intervention, as well as by the GP centric nature and single payer structure of the Italian health system. Other health system leaders should evaluate the appropriate targets (e.g., GPs, specialists) and consider the extent to which strong provider engagement is likely within their own local context. Beyond interventions targeting prescribers, educating patients regarding the risks of PIM use can increase motivation to deprescribe hazardous drugs and should be considered as another mechanism for reducing PIM use [41]. A majority of older adults would like to reduce the number of medications they are taking [42]. Further research is needed to understand the extent to which multimodal interventions that simultaneously target both patients and prescribers could produce synergistic reductions in rates of PIM exposure and associated adverse events.

## Conclusion

To date, our analysis is the first that has attempted to estimate the relationship between a multifaceted local health system wide general practitioner focused intervention to reduce PIMs and changes in unplanned hospitalization rates among a locality’s older adult population. Our research suggests that interventions to reduce PIMs in older adults can also decrease unplanned hospitalization rates. This reduction was found to be the most pronounced during the time period of the intervention, presumably yielding clinical benefits for the patients, as well as economic savings for Parma LHA. In the face of aging populations and escalating health care costs, multimodal educational outreach interventions to reduce PIM exposure hold promise for health systems seeking to improve health outcomes and reduce the utilization of high-cost inpatient care.

## Supplementary Information

**Additional file 1.**

## Data Availability

The data that support the findings of this study are available from the Local Health Authority of Parma, but restrictions apply to the availability of these data, which were used under license for the current study, and so are not publicly available.

## Abbreviations

PIMs: Potentially inappropriate medications
LHA: Local Health Authority
Q1: First quarter
Q2: Second quarter
Q3: Third quarter
Q4: Fourth quarter
CCDGs: Chronic condition drug groups
IRRs: Incidence rate ratios
CI: Confidence interval
