# Supplementary material for: Reduction in unplanned hospitalizations associated with a physician focused intervention to reduce potentially inappropriate medication use among older adults: a population-based cohort study
Source: BMC Geriatr. 2021 Mar 31;21:218. doi: 10.1186/s12877-021-02172-3 (PMC8011227; doi:10.1186/s12877-021-02172-3)
Supplement: Supplementary file 1 — Additional file 1. [file 12877_2021_2172_MOESM1_ESM.docx]

**Supplemental Material**

Reduction in Unplanned Hospitalizations Associated with a Physician Focused Intervention to Reduce Potentially Inappropriate Medication Use Among Older Adults: A Population-Based Cohort Study

Alcusky M,^1^ Thomas RB,^2^ Jafari N,^3^ Keith SW,^4^ Kee A,^2^ Del Canale S,^5^ Lombardi M,^5^ Maio V^2^

^1^Department of Population and Quantitative Health Sciences, University of Massachusetts Medical School, Worcester, MA, USA

^2^Jefferson College of Population Health, Thomas Jefferson University, Philadelphia, PA, USA

^3^Center for Research in Medical Education and Health Care, Sidney Kimmel Medical College, Thomas Jefferson University, Philadelphia, PA, USA

^4^Division of Biostatistics, Department of Pharmacology and Experimental Therapeutics, Sidney Kimmel Medical College, Thomas Jefferson University, Philadelphia, PA, USA

^5^Azienda Unità Sanitaria Locale di Parma (Local Health Authority of Parma), Parma, Italy

| Additional File 1. Diagnosis Based Algorithms to Identify Hospitalizations for Potential NSAID and Digoxin Related ADEs | |
| --- | --- |
| Clinical Condition | ICD-9 CM Codes |
| Atrial fibrillation/flutter^1^ (first position) | 42731, 42732 |
| Other arrhythmias (first position) | 426, 427, 785.0, 785.1, 798 |
| Ischemic stroke^2^ (first position) | 43301, 43311, 43321, 43331, 43381, 43391, 43401, 43411, 43491, 436 |
| Transient ischemic attack (first position) | 435.0, 435.1, 435.2, 435.3, 435.8, 435.9 |
| Extracranial bleeding^3^ (first position or second with specified other code in first position) | In primary position alone:  5310, 5312, 5314, 5316, 5320, 5322, 5324, 5326, 5330, 5332, 5334, 5336, 5340, 5342, 5344, 5346, 53501, 53511, 53521, 53531, 53541, 53551, 53561, 53783, 4560, 45620, 5307, 53082, 5780, 4552, 4555, 4558, 56202, 56203, 56212, 56213, 56881, 5693, 56985, 5781, 5789, 59381, 5997, 6238, 6262, 6266, 4230, 4590, 56881, 7191, 7847, 7848, 7863  In primary position, with above code in secondary position:  5311, 5313, 5315, 5317, 5319, 5321, 5323, 5325, 5327, 5329, 5331, 5333, 5335, 5337, 5339, 5341, 5343, 5345, 5347, 5349, 53500, 53510, 53520, 53530, 53540, 53550, 53560, 455, 56200, 56201, 56210, 56211, 5301, 2800, 2851, 2859, 79092 |
| Acute myocardial infarction^4^ (first or second position) | 41001, 41011, 41021, 41031, 41041, 41051, 41061, 41071, 41081, 41091 |
| Venous thromboembolism^5^ (first or second position) | 41511, 41519, 45111, 45119, 4512, 4519, 4531, 4532, 4534, 45341, 45342, 4538, 4539 |
| Systemic embolism^6^ (first position) | 445, 445 |
| Heart Failure^7^ (first position) | 398.91, 402.01, 402.11, 402.91, 404.01, 404.03, 404.11, 404.13, 404.91, 404.93, 428.0, 428.1, 428.20, 428.21, 428.22, 428.23, 428.30, 428.31, 428.32, 428.33, 428.40, 428.41, 428.42, 428.43, 428.9 |
| Hypertension^7^ (first position) | 362.11, 401.0, 401.1, 401.9, 402.00, 402.01, 402.10, 402.11, 402.90, 402.91, 403.00, 403.01, 403.10, 403.11, 403.90, 403.91, 404.00, 404.01, 404.02, 404.03, 404.10, 404.11, 404.12, 404.13, 404.90, 404.91, 404.92, 404.93, 405.01, 405.09, 405.11, 405.19, 405.91, 405.99, 437.2 |
| Digitalis poisoning (any position) | 972.1 |
| Acute renal failure (first position) | 5724, 5800, 5804, 58089, 5809, 584, 5824, 7912, 7913, 586 |
| Chronic renal failure^8^ (first position) | 582, 583, 585, 586, 587 |
| Electrolyte disorders (first position) | 276 |

| Additional File 2. Sensitivity Analyses Varying the Lag Period for the Association between a Quality Improvement Initiative to Reduce PIM Use and Quarterly Unplanned Hospitalization Rates | | | |
| --- | --- | --- | --- |
|  | Estimate^a^ | IRR^a^ (95% CI) |  |
| 0.5 Year Lag Period | | |  |
| Time (per quarter)^b^ |  |  |  |
| Pre-Intervention | -0.007 | 0.993 (0.991, 0.995) |  |
| Intervention | -0.017 | 0.983 (0.980, 0.986) |  |
| Post-Intervention | -0.003 | 0.997 (0.995, 0.998) |  |
| 1.5 Year Lag Period | | |  |
| Time (per quarter)^b^ |  |  |  |
| Pre-Intervention | -0.008 | 0.992 (0.990, 0.994) |  |
| Intervention | -0.018 | 0.982 (0.978, 0.985) |  |
| Post-Intervention | 0.001 | 1.001 (0.999, 1.003) |  |
| ^a^Estimates and incidence rate ratios for quarterly changes in unplanned hospitalization rates during the specified time period from a multivariable longitudinal negative binomial GEE model ^b^Time represented in the model as the piecewise linear truncated power basis spline. The pre-intervention, intervention, and post-intervention estimates were calculated from the linear combination of coefficients for three time trend variables.  IRR = Incidence Rate Ratio, CI = Confidence Interval, PIM = Potentially Inappropriate Medication, GEE = Generalized Estimating Equations | | | |

| Additional File 3. Association between a Quality Improvement Initiative to Reduce PIM Use and Quarterly Unplanned Hospitalization Rates for Diagnoses Representing Potential NSAID and Digoxin Related ADEs | | | |
| --- | --- | --- | --- |
|  | Estimate^a^ | IRR^a^ (95% CI) |  |
| Time (per quarter)^b^ |  |  |  |
| Pre-Intervention | -0.012 | 0.988 (0.985, 0.991) |  |
| Intervention | -0.015 | 0.986 (0.981, 0.990) |  |
| Post-Intervention | -0.010 | 0.990 (0.987, 0.993) |  |
| ^a^Estimates and incidence rate ratios for quarterly changes in unplanned hospitalization for diagnoses representing potential NSAID and digoxin related ADEs rates during the specified time period from a multivariable longitudinal negative binomial GEE model  ^b^Time represented in the model as the piecewise linear truncated power basis spline. The pre-intervention, intervention, and post-intervention estimates were calculated from the linear combination of coefficients for three time trend variables.  IRR = Incidence Rate Ratio, CI = Confidence Interval, PIM = Potentially Inappropriate Medication, ADE = Adverse Drug Event, NSAID = Non-Steroidal Anti-Inflammatory Drug, GEE = Generalized Estimating Equations | | | |

| Additional File 4. Associations between Patient Characteristics, Intervention Period, and Hospitalization Rates | | | |
| --- | --- | --- | --- |
|  | Estimate^a^ | IRR (95% CI)^a^ |  |
| Intercept | -8.394 |  |  |
| Age (per year) | 0.062 | 1.064 (1.063, 1.065) |  |
| Women | -0.357 | 0.700 (0.688, 0.711) |  |
| Location |  |  |  |
| Plain | Ref |  |  |
| Hill | -0.013 | 0.987 (0.970, 1.005) |  |
| Mountain | -0.108 | 0.898 (0.874, 0.922) |  |
| CCDG (per each) | 0.068 | 1.071 (1.069, 1.072) |  |
| Season |  |  |  |
| Winter | Ref |  |  |
| Fall | -0.040 | 0.961 (0.948, 0.975) |  |
| Summer | -0.117 | 0.890 (0.876, 0.903) |  |
| Spring | -0.046 | 0.955 (0.941, 0.969) |  |
| Time (per quarter)^a^ |  |  |  |
| Pre-Intervention^b^ | -0.007 | 0.993 (0.991, 0.995) |  |
| Intervention^b^ | -0.018 | 0.982 (0.979, 0.985) |  |
| Post-Intervention^b^ | -0.001 | 0.999 (0.997, 1.001) |  |
| ^a^Estimates and incidence rate ratios for quarterly changes in unplanned hospitalization rates during the specified time period from a multivariable longitudinal negative binomial GEE model  ^b^Time represented in the model as the piecewise linear truncated power basis spline. The pre-intervention, intervention, and post-intervention estimates were calculated from the summation of coefficients for three time trend variables.  IRR = Incidence Rate Ratio, CI = Confidence Interval, CCDG = Chronic Condition Drug Group, GEE = Generalized Estimating Equations | | | |

**References**

1. Jensen PN, Johnson K, Floyd J, Heckbert SR, Carnahan R, Dublin S. A systematic review of validated methods for identifying atrial fibrillation using administrative data. Pharmacoepidemiol Drug Saf. 2012 Jan;21 Suppl 1:141-7.

2. Kumamaru H, Judd SE, Curtis JR, Ramachandran R, Hardy NC, Rhodes JD, Safford MM, Kissela BM, Howard G, Jalbert JJ, Brott TG, Setoguchi S. Validity of claims-based stroke algorithms in contemporary medicare data: reasons for geographic and racial differences in stroke (REGARDS) study linked with medicare claims. Circ Cardiovasc Qual Outcomes. 2014;7:611–9.

3. Cunningham A, Stein CM, Chung CP, Daugherty JR, Smalley WE, Ray WA. An automated database case definition for serious bleeding related to oral anticoagulant use. 2011;(March):560-566.

4. Kiyota Y, Schneeweiss S, Glynn RJ, Cannuscio CC, Avorn J, Solomon DH. Accuracy of medicare claims-based diagnosis of acute myocardial infarction: Estimating positive predictive value on the basis of review of hospital records. *Am Heart J*. 2004;148(1):99-104.

5. White RH, Garcia M, Sadeghi B, et al. Evaluation of the predictive value of ICD-9-CM coded administrative data for venous thromboembolism in the United States. Thromb Res. 2010;126(1):61-67.

6. Coleman CI, Peacock WF, Bunz TJ, Alberts MJ. Effectiveness and Safety of Apixaban, Dabigatran, and Rivaroxaban Versus Warfarin in Patients with Nonvalvular Atrial Fibrillation and Previous Stroke or Transient Ischemic Attack. *Stroke*. 2017;48(8):2142-2149.

7. Chronic Conditions Data Warehouse. Condition Categories. Accessed January 26, 2021. <https://www2.ccwdata.org/web/guest/condition-categories>

8. Winkelmayer WC, Schneeweiss S, Mogun H, Patrick AR, Avorn J, Solomon DH. Identification of individuals with CKD from medicare claims data: A validation study. *Am J Kidney Dis*. 2005;46(2):225-232.
